# A Continuity in Logical Development: Domain-General Disjunctive Inference by Toddlers

**DOI:** 10.1162/opmi_a_00148

**Published:** 2024-06-28

**Authors:** Nicolò Cesana-Arlotti, Justin Halberda

**Affiliations:** Department of Psychology, Yale University, New Haven, CT, USA; Department of Psychological and Brain Sciences, Johns Hopkins University, Baltimore, MD, USA

**Keywords:** cognitive development, disjunctive syllogism, word learning, social learning, negation, logic

## Abstract

Children grow up surrounded by opportunities to learn (the language of their community, the movements of their body, other people's preferences and mental lives, games, social norms, etc.). Here, we find that toddlers (N = 36; age range 2.3–3.2 years) rely on a logical reasoning strategy, Disjunctive Inference (i.e., A OR B, A is ruled out, THEREFORE, B), across a variety of situations, all before they have any formal education or extensive experience with words for expressing logical meanings. In learning new words, learning new facts about a person, and finding the winner of a race, toddlers systematically consider and reject competitors before deciding who must be the winner. This suggests that toddlers may have a general-purpose logical reasoning tool that they can use in any situation.

## INTRODUCTION

The capacity to think using abstract logical operations is at the core of some of our species’ most powerful abilities. By representing logical relations between hypotheses, we can think not just about the way things are, but also about the ways things cannot be, and draw inferences about the way they must be. This capacity is seminal for both professional scientific reasoning and reasoning in daily life. We do this, for example, when we use Disjunctive Inference (e.g., EITHER the sun OR the earth is at the center of the planets’ orbits; it is NOT the earth, because this would require implausibly complicated orbits; THEREFORE, the sun is at the center of the planets’ orbits).

Even more powerfully, the human aptitude for domain-general learning, planning, and decision-making rests on a foundation of logical representations that allow us to reason about observations from any domain, from physics to knitting, using the same domain-general inference schemas (Fodor, 1979; Tarski & Corcoran, 1986). In this sense, domain-general logical abilities are a multi-use tool – ready to be deployed for new challenges in any domain. Where does this logical ability come from—is it a hard-won accomplishment of educated adults, or is it within the grasp even of young children? Suggestive of a domain-general ability to reason using Disjunctive Inference,^1^ a number of studies have investigated children’s abilities to reason using disjunction and/or negation (Austin et al., 2014; Cesana-Arlotti et al., 2018, 2022; de Carvalho et al., 2021; De Villiers & Flusberg, 1975; Ekramnia et al., 2021; Feiman et al., 2017, 2022; Hochmann & Toro, 2021; Markman & Wachtel, 1988; Reuter et al., 2018). Of course, the more numerous and varied the tasks that children succeed with, the more likely it is that success rests on a domain general ability. But, previous work remains open to a variety of deflationary accounts that do not implicate Disjunctive Inference: such as mapping novelty-to-novelty (mapping an unfamiliar-name to an unfamiliar-object, Pomiechowska et al., 2021; mapping an unfamiliar-voice to an unfamiliar-face; Ekramnia et al., 2021; Moher et al., 2010), avoidance (avoid the empty cup; avoid the causally-irrelevant object; Feiman et al., 2022) or contradictory mapping by negation (“look that is a non-bamoule!” de Carvalho et al., 2021). Because no single bias would seem to capture performance in all of these studies, this lends some credence to the possibility that a more domain general inference is called on across these cases. In addition, a handful of studies have focused on whether children as young as 2.5 years can deploy the foundational logical schema Disjunctive Inference while including controls for avoidance. However, these studies have only tested its use within a single domain – the tracking of hidden objects, and so cannot address the issue of domain generality (Gautam et al., 2021; Grigoroglou et al., 2019; Mody & Carey, 2016).

In a complementary line of work, Cesana-Arlotti and colleagues explored Disjunctive Inference in preverbal infants with tasks involving ambiguity about object identity (Cesana-Arlotti et al., 2018, 2022). Crucially, in one study, 14-month-old infants learned the preference of an agent reaching for a goal-object whose identity was concealed—it had to be inferred using Disjunctive Inference (Cesana-Arlotti et al., 2020). Infants’ success in this task can be explained with a form of logical inference about an agent’s preference (i.e., the agent prefers the toy car OR she prefers the ball; she avoided the ball; THEREFORE, she prefers the toy car). Alternatively, infants may first make a logical inference about the identity of the hidden object (i.e., the hidden object is the toy car OR the ball; the hidden object is a different object than the ball; THEREFORE, the hidden object is the toy car) and only afterward attribute the preference. Hence, this finding hints at a possible domain generality of infants’ logical representation, but, clearly, targeted testing of domain generality is still required.

Here, rather than testing children in a wide variety of contexts, we constructed a simple two-alternative forced-choice procedure, implemented in three different domains, and relied on toddlers’ active rejection of a competitor as a measure of their engagement in Disjunctive Inference across distinct domains.

What would it mean for children to have a domain-general understanding of Disjunctive Inference? At least, with adults, it is transparent that we know the words *or* and *not* and we can apply them to any domain – so we exhibit domain generality. However, the link between nonverbal Disjunctive Inferences and children’s understanding of verbal negation is not well understood. While verbal negation is powerful because it can apply to any domain (“not the ball,” “not Mommy,” “not loud”), it is unknown whether children’s early understanding of verbal negation in fact implicates domain generality. Toddlers respond to sentences that explicitly deny the presence of an object at a location (i.e., “the ball is not in the bucket”) by avoiding the location and searching elsewhere (Austin et al., 2014; Feiman et al., 2017). But this might reflect avoidance based on domain-specific representations (e.g., “*avoid that location*” or “*that location is empty*”), rather than the logical operation of elimination that can support domain-general inference. That is, to be a domain-general inference requires that the same inference schema be used, irrespective of the reason for rejecting a competitor. What we seek is a behavioral signature that this rejection is a common strategy across domains (and that the resulting elimination promotes high confidence that the alternative target is the correct choice). Here, we investigate the behavioral signature of a “double-check” of the competitor object as a metric for engagement in the rejection that is central to Disjunctive Inference and a lack of redundant saccades and a rapid mapping to the target as metrics for the increased certainty that drives target selection.

We asked whether toddlers could perform Disjunctive Inferences in three tasks that were identically structured except for the content toddlers were required to reason over, which varied widely (see Supplementary Note 1). The Word-Learning task asked toddlers to identify familiar vs. unfamiliar objects (e.g., “Point at the ball/dax” (Markman & Wachtel, 1988)) (n = 12; age range 2.3–2.8 years); the Social-Learning task required toddlers to identify animals based on their previously learned (i.e., familiar) versus novel (unfamiliar) preferences (e.g., “I like taking walks”) (n = 12; age range 2.5–3.4 years); and, the Explicit Negation task had toddlers identify the winner of a race using a positive or negative assertion (i.e., positive “The winner is the bird,” negative “The winner is not the bird”, Horn, 2001) (n = 12; age range 2.4–3.2 years). The set-up for each game was similar, and quite simple. In each task, across many trials, toddlers saw two options and had to choose just one of them. For example, in the Word Learning task, toddlers might see a ball and an unfamiliar object, and hear “Point at the dax!” If toddlers infer the right choice by rejecting the wrong one, they have performed Disjunctive Inference (e.g., “dax” must refer to one object or the other; “dax” does not refer to the ball; therefore, “dax” must refer to the unfamiliar object).

Performing Disjunctive Inference is not, however, the only way to succeed in our tasks. For example, toddlers might succeed on unfamiliar trials in the three tasks without using Disjunctive Inference, simply by positively seeking an object that contrasts with the competitor – e.g., they need only “map novelty to novelty” (a positive strategy that does not predict double-checking the familiar object; Halberda, 2006; Mervis et al., 1994). How can we ascertain whether toddlers use a logical inference in our tasks?

**Figure 1.**
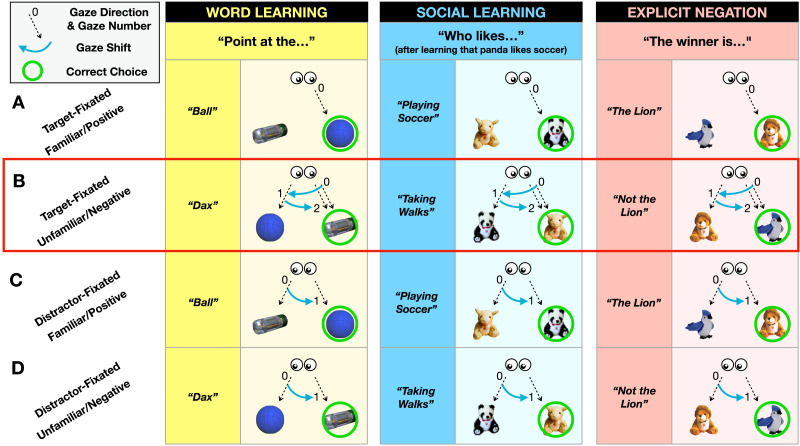
Predictions of looking patterns and pointing in the three tasks. Across tasks, toddlers saw two objects/animals that were either familiar or unfamiliar. On some trials they heard *Familiar*/*Positive prompts*: a familiar name in Word Learning, a preference known to be associated with a particular animal in Social Learning, an assertion stating who was the winner in Explicit Negation. On other trials, toddlers heard *Unfamiliar*/*Negative prompts*: an unfamiliar name in Word Learning, an unfamiliar preference in Social Learning, a negation stating who was NOT the winner in Explicit Negation. We predicted distinct looking patterns as a function of prompt type and whether the object fixated at the time of the prompt was the target or distractor. (A) *Target-Fixated Familiar*/*Positive trials*: toddlers need not exclude the distractor to identify the target (e.g., they can recognize that “ball” refers to the ball), and thus are unlikely to shift gaze toward the distractor before pointing. (B) *Target-Fixated*, *Unfamiliar*/*Negative trials*: by hypothesis, toddlers must mentally exclude the distractor to infer the target (e.g., “dax” does not refer to the ball; therefore, “dax” must refer to the unfamiliar object). Thus, they are more likely to shift gaze to the distractor and then back to the target before pointing. (C) *Distractor-Fixated*, *Familiar*/*Positive trials*: toddlers can directly mentally exclude the fixated distractor (e.g., “ball” does not refer to the unfamiliar object); thus, they are likely to shift gaze straight to the target, then point at it right away. (D) *Distractor-Fixated*, *Unfamiliar*/*Negative trials*: toddlers can directly mentally exclude the fixated distractor and infer the target (e.g., “dax” does not refer to the ball; therefore, it must refer to the unfamiliar object). By hypothesis, they are likely to shift gaze to the target, then point right away.

We predicted that if toddlers actively reason through the rejection of the competitor object (e.g., “dax” does not refer to the ball), as adults and older children do, then they may show an increased tendency to shift their gaze to the competitor as they think through this rejection (Halberda, 2006). We recorded toddlers’ eye movements throughout each trial. Inspired by work in adults and preschoolers (Halberda, 2006), we formalized the following prediction: if toddlers were already fixating the unfamiliar target object at the moment of hearing the unfamiliar name (“dax”), they should show an increased tendency to “double-check” the familiar option (as they mentally reject it: “that is a ball, so it cannot be a dax”) before bringing their eyes back to and pointing at the unfamiliar target as the winner. In contrast, fewer double-checks are predicted on *Target-Fixated* familiar name trials (e.g., looking at the ball while hearing “ball”), where Disjunctive Inference is *not* required; on these trials, children should tend to remain fixated on the target (ball) and immediately point, as they do not need to mentally exclude the other object to identify the ball. Similar predictions held for the other two tasks (Social-Learning and Explicit Negation). Figure 1 details each trial type in the three tasks (children saw multiple examples, intermixed, of each trial type with a wide variety of objects/animals).

In summary, there are at least two ways to succeed in our tasks: Disjunctive Inference or a positive search for an object that contrasts with the competitor (e.g., an unfamiliar object, an unfamiliar puppet, or a non-lion animal, Figure 1). Crucially, only Disjunctive Inference predicts that participants will be more likely to perform a double-check on *Target-Fixated Unfamiliar*/*Negative trials*. Previous research found precisely this eye-movement pattern in adults and preschoolers’ reasoning through Disjunctive Inference (Halberda, 2006). However, it is unknown whether younger word learners deploy Disjunctive Inference too, and whether this logical capacity already extends across multiple cognitive domains. In our work here with toddlers, we are predicting a developmental *continuity* – toddlers should be old enough to succeed on all three tasks and we expect that they (like preschoolers and adults) will succeed on *Target-Fixated Unfamiliar*/*Negative trials* by visually double-checking the familiar competitor in order to explicitly reject it before correctly mapping the *Unfamiliar*/*Negative prompt* to the target object/animal. If toddlers already have access to domain-general Disjunctive Inference, then the double-checking should emerge across different tasks.

## METHOD

### Participants and Trial Exclusions

The sample size for these experiments (N = 12, each) was chosen based on a previous experiment with preschoolers that measured eye movements (i.e., the tendency to double-check the competitor) to investigate Disjunctive Inference in word learning (Halberda, 2006). The preschooler experiment yielded a large effect size d = 0.98. A power analysis (Faul et al., 2007) indicated that 12 participants would be sufficient to provide >80% statistical power to detect this large effect. Additional pilot studies in the lab suggested that toddlers would be near-ceiling at pointing to the correct answer across all trial types in our tasks. Please note that our study was designed to test for a continuity in development, not to investigate developmental change – which might have required a larger sample size and a wider age range.

Similarly, an alternative approach for establishing domain generality would be to look for consistent patterns of individual differences across the three tasks. This too would likely require a larger sample size. Note, however, that our pilot studies suggest that toddlers will be near-ceiling across our three tasks—and this is precisely what we might expect if, as hypothesized, Disjunctive Inference is a basic domain general inference shared by all toddlers. Given the consistency of our methods across our three tasks, it is also possible to consider our tasks to be three between-subjects conditions in a single experiment with 36 participants.

This study was approved by the Johns Hopkins University Homewood Internal Review Board and the Harvard Internal Review Board. Parents gave written informed consent, and toddlers received a small gift (T-shirt, stuffed animal, book) for their participation.

Individual test trials were excluded from analysis for: looking at the caregiver before answering (less than 1%), fussiness (less than 1%), reaching for an object before labeling (2%), reaching for both objects (less than 1%), refusing to answer the prompt (less than 1%), experimenter’s error (1.5%) or the toddler asking for a specific animal to win the race (less than 1%).

Twenty-six additional children were tested across the three experiments, but not included in the final sample due to fussiness (12), repeated refusal to point (3), equipment failure (e.g., participant’s gaze was not visible in the recording (5)), or experimenter error (e.g., the experimenter used incorrect prompts or trial order, (6)). Additional participants (7) completed the task, but were excluded from the analyses since they lacked valid trials in at least one of the four conditions (*Target-Fixated*, *Familiar*/*Positive*; *Target-Fixated*, *Unfamiliar*/*Negative*; *Distractor-Fixated*, *Familiar*/*Positive*; *Distractor-Fixated*, *Unfamiliar*/*Negative*) that was a prerequisite for the double-check analysis. These happened by chance because the experimenter exerted no active control on which object a participant was fixating at the time of the prompt. Importantly, success in the pointing results remains unchanged if these seven children are included in the sample (n = 43, see Supplementary Note 2).

### Procedures

For all three tasks, two objects were presented on each trial, and a verbal prompt cued toddlers to choose one object (Figure 1, see Supplementary Note 1 for the details of the three tasks’ procedure). We focused on two factors. First, the target object could be indicated by either a *Familiar*/*Positive prompt* or an *Unfamiliar*/*Negative prompt*. Second, by their own free looking, toddlers either happened to be looking at the target object or at the distractor object at the moment of hearing the prompt (or, rarely, at neither). This resulted in four trial types of interest, which are documented for each task in Figure 1. For each experiment, approximately half of the trials involved two familiar/positive alternatives (e.g., two familiar target objects with familiar names; two familiar animals with familiar preferences; two familiar animals who had previously been in at least one race) while half of the trials involved one familiar/positive alternative and one unfamiliar/negative alternative (e.g., one familiar target object with a familiar name and one unfamiliar target object; one familiar animal with a familiar preference and one unfamiliar animal; one familiar animal who had previously participated in a race and one new animal who had not previously participated in a race). When a novel item was included on a trial, approximately half of the time it was the target and half of the time it was the distractor.

First, we briefly sketch the trial structure for both *Familiar*/*Positive trials* (which served as an internal control because no Disjunctive Inference was required) and *Unfamiliar*/*Negative trials* (which required Disjunctive Inference). For *Familiar/Positive trials*, see row A in Figure 1. In the Word-Learning task, toddlers are already familiar with the word “ball”, and so can readily point to the ball when told “Point at the ball.” In the Social-Learning task toddlers were already familiar with panda and had previously learned that panda likes soccer (from the training trials), and so can point at the panda when asked who likes soccer. And in the Explicit Negation task toddlers simply heard the assertion that “the winner is the lion”. Importantly, none of these trial types required toddlers to reason using Disjunctive Inference.

For examples of *Unfamiliar*/*Negative trials*, see row B in Figure 1. In the Word-Learning task toddlers saw a familiar object (e.g., ball) and a novel object, and were asked to “Point at the [novel label, e.g., “dax”]. In the Social-Learning task toddlers saw a familiar animal whose preference they had already learned (e.g., panda), as well as a novel animal whose preference they were unaware of, and then were asked “Who likes [novel activity, e.g., “taking walks”]?”. And in the Explicit Negation task, toddlers heard the winner indicated via negation (e.g., “the winner is NOT the lion.”). Toddlers could solve these trial types by either positively mapping novel/negative information to the novel/contrasting item (i.e., Map-Novelty-to-Novelty) or by reasoning using Disjunctive Inference.

For all trial types, toddlers saw multiple trials with different animals, objects, or preferences.

To understand our behavioral predictions, consider the predicted looking for *Target-Fixated* and *Distractor-Fixated Familiar/Positive trials* (row A & C, Figure 1). Toddlers were free to look at whichever object they liked during the period from the objects’ appearance until the prompt was spoken. On most trials (n = 78%), toddlers fixated each object during this time, and then they happened to be fixating either the target object (row A, Figure 1) *or* the distractor object (row C, Figure 1) at the moment of the verbal prompt (e.g., “point at the ball”). When toddlers happened to be fixating the target (e.g., ball) at the time of the prompt (row A, Figure 1), we predict that many of the trials will involve zero shifts in gaze. That is, toddlers can recognize the target and can directly point at it (in fact, it doesn’t matter so much if they occasionally shift gaze on these trials, because our prediction is really a relative one – that there will be more shifts in gaze on *Target-Fixated Unfamiliar*/*Negative trials* (row B, Figure 1) then on *Target-Fixated Familiar*/*Positive trials* (row A, Figure 1).

On the *Distractor-Fixated Familiar*/*Positive trials* (row C, Figure 1), we predict one shift in gaze as children will reject the distractor as being the target of the prompt (e.g., “point at the ball”), shift fixation to the target object (e.g., ball) recognize it and point (0 → 1 in row C, Figure 1).

Next, consider those trials where the target prompt is *Unfamiliar/Negative* (e.g., “point at the dax”), and when toddlers happen to be fixating the distractor object (e.g., ball) at the moment of hearing the prompt (row D, Figure 1). We predict one shift in gaze (i.e., away from the distractor object, towards the target object, followed by a point; 0 → 1 in row D, Figure 1). This is because the toddlers can reject the distractor based on the prompt, and having rejected the distractor they can infer that the other object (e.g., the novel object) must be the target (that is, they can perform Disjunctive Inference).

What about those *Unfamiliar*/*Negative trials* where toddlers happen to be fixating the target (e.g., novel object) at the time of hearing the prompt (row B, Figure 1)? Can they directly and positively decide that the fixated object must be the target? This would be to positively map-novelty-to-novelty and to not perform Disjunctive Inference. We predict instead that, on these *Target-Fixated Unfamiliar*/*Negative trials*, toddlers will perform Disjunctive Inference and will reject the distractor object (e.g., ball) as a possible target for the prompt. We predict that the mental act of rejecting the distractor object will be linked to an increased likelihood of making a shift in gaze to it and then back (0 → 1 → 2 in row B, Figure 1). Note, a shift in gaze is not required on all of these trials, because the toddler might simply carry out Disjunctive Inference in their minds, without a shift in gaze (e.g., *The winner is not the lion*; *he is over there*; *so the winner must be this bird I am looking at*). As we noted above, our prediction is a relative one: that toddlers will be more likely to make this shift in fixation on *Target-Fixated Unfamiliar*/*Negative trials* (row B, Figure 1) then on *Target-Fixated Familiar*/*Positive trials* (row A, Figure 1). Such a difference will serve as evidence that toddlers have succeeded by working through Disjunctive Inference. Finally, we probed the domain-generality of the toddlers’ inferential strategy by comparing the probability of the double-check across different tasks. Previous work has tested the domain-generality of adults’ reasoning by comparing their performance across tasks tapping on distinct cognitive domains (Beaman, 2002; Cosmides, 1989; Johnson-Laird et al., 1972). If toddlers recruit different strategies to solve our three tasks, we expect that the increase in the probability of the double-check in the Target-Fixated Unfamiliar/Negative trials relative to the Familiar positive trials will be different across the tasks. On the contrary, the absence of an interaction effect between tasks and prompt types would suggest that the toddlers use the same inference in all of them.

## RESULTS

We started by asking whether preschoolers can successfully infer the target of the experimenter’s prompt in both the *Familiar*/*Positive* and in the *Unfamiliar*/*Negative* conditions of each task. For each task and each participant, we computed the proportion of correct choices in each test condition (*Familiar*/*Positive* vs. *Unfamiliar*/*Negative*). We ran Sign tests to compare the proportion of correct choices against chance (50%). We found that children performed near-ceiling on all trial types across the three tasks: *Word Learning*, *Unfamiliar Name*, *M* = 84% correct, *SD* = 17%, *Familiar Name*, *M* = 96%, *SD* = 7%; *Social Learning*, *Unfamiliar Preference*, *M* = 95%, *SD* = 10%, *Familiar Preference*, *M* = 95%, *SD* = 6%; *Explicit Negation*, *Positive Prompt*, *M* = 95%, *SD* = 10%, *Negative Prompt*, *M* = 98%, *SD* = 6%. These values were all well above chance (all two-tailed Sign test *p*’s < .001, n = 12).

### Toddlers’ Double-Check Analysis

Our main question is not whether the children would succeed in the three tasks. Indeed, we have plenty of evidence that toddlers can find the referent of novel names (Halberda, 2003; Lewis et al., 2020; Spiegel & Halberda, 2011) or respond to verbal negation (Austin et al., 2014; Feiman et al., 2017). Our target question is what computations support this success across the different tasks: do toddlers use Disjunctive Inference in the three tasks, as adults do in Word Learning and Explicit Negation tasks (see Halberda, 2006). To test the predictions of our hypothesis of continuity in logical development (see Figure 1), we relied on a conservative criterion and measured when *exactly one* double-check was executed on trials where toddlers chose correctly during the decision phase (i.e., the temporal window starting from the end of the experimenter’s question until the child pointed to or grasped one of the two objects)^2^.

**Figure 2.**
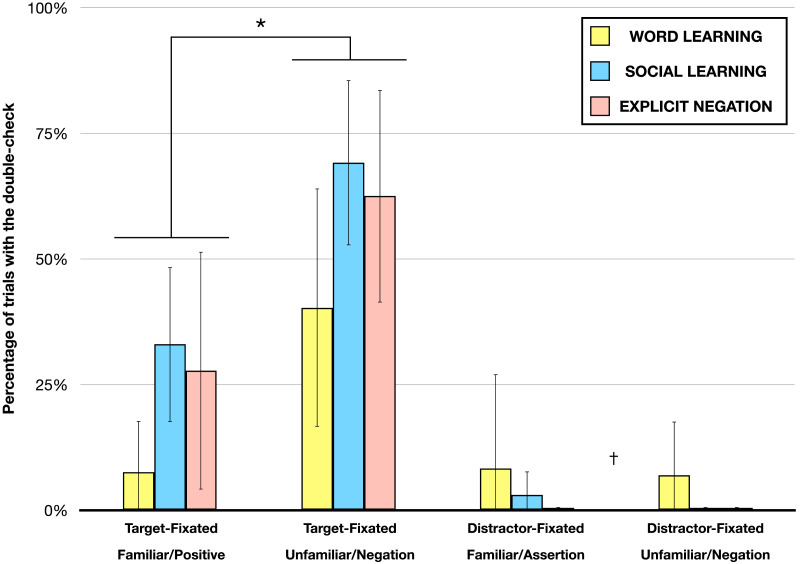
Percentage of trials with one double-check as a function of prompt type and object fixated. Bars reflect the percentage of trials with one double-check prior to object choice, error bars represent 95% confidence intervals. Across all three tasks, the percentage of trials with one double-check was higher when toddlers were fixating the target and heard an *Unfamiliar*/*Negative prompt*, consistent with the Disjunctive Inference. Bayes Factor tests reveal that an effect of prompt type on *Target-Fixated trials* is >50 times *more* likely than no effect (*), and that an effect of prompt type on *Distractor-Fixated trials* is >50 times *less* likely than no effect (†).

An appropriate statistical approach to answer our question is to fit mixed effects logistic regression models predicting the presence of the double-check in the *Target-Fixated trials* (n = 195), using the r package lm4. Each model had a fixed effect for Task (i.e., Word-Learning vs. Social-Learning vs. Explicit-Negation) and a random intercept for participant, nested in Task. The first model “H0” had no additional effects. The second model “H1” had a fixed effect for Prompt Type (i.e., *Familiar*/*Positive* vs. *Unfamiliar*/*Negative*). The third model “H2” had a fixed effect for Prompt Type and an interaction effect between Prompt Type and Task. To compare pairs of models we computed a Bayes factor (Linzen & Gallagher, 2017), which is a ratio of the likelihoods of the observed data under two different models. The Bayes Factor between two models was approximated by the Bayes Information Criteria of the models (Wagenmakers, 2007) (See Supplementary Note 3 for details about the analysis). The Bayesian analysis of double-checks on *Target-Fixated trials* revealed that the data are 73 times more likely under H1 than H0: in the *Target-Fixated trials* the double-check is more probable in the *Unfamiliar*/*Negative* condition than in the *Familiar*/*Positive* one, (*Unfamiliar*/*Negative*, *M* = 57%, *SD* = 33%; *Familiar*/*Positive* = 23%, *SD* = 28%). Furthermore, the analysis revealed that the data are 149 times more likely under H1 than H2: that is, there is no interactions and the three tasks resulted in a similar pattern of results (see Figure 2). This result was confirmed by an ANOVA (Type II Wald chi square tests, r package car) that detected a main effect of Prompt Type (X^2^ (1, N = 195) = 21.07, *p* < .001), a main effect of Task (*Word-learning*, *M* = 24%, *SD* = 32%; *Social-Learning*, *M* = 51%, *SD* = 30%; *Explicit-Negation*, *M* = 45%; *SD* = 38%; X^2^ (2, N = 195) = 12.88, *p* < .01), but no interaction effect (X^2^ (2, N = 195) = 0.59, *p* > .05). Planned follow-up pairwise comparisons (r package emmeans) corroborated the effects of Prompt Type in each Task: *Word-learning* (*Unfamiliar*, *M* = 40%, *SD* = 37%; *Familiar*, *M* = 8%, *SD* = 16%; *p* < .01), *Social-learning* (*Unfamiliar*, *M* = 69%, *SD* = 25%; *Familiar*, *M* = 33%, *SD* = 24%; *p* < .01), *Explicit-Negation* (*Unfamiliar*, *M* = 62%, *SD* = 33%; *Familiar*, *M* = 28%, SD = 36%; *p* < .05).

Next, we considered the scanning pattern that occurred on both *Distractor-Fixated Familiar*/*Positive trials* and *Distractor-Fixated Unfamilar*/*Negative trials*. On these trials (n = 184), by our hypothesis, children should have no reason to double-check. They should simply reject the distractor, move their eyes to the target, and indicate their choice. Crucially, by our hypothesis there should be no difference in the probability of double-checks on *Unfamiliar*/*Negative trials* versus *Familiar*/*Positive trials* in cases where children were fixating the distractor. This would demonstrate that the *Unfamiliar*/*Negative prompts* do not simply lead children uniformly to increase shifts in gaze. To test this, we applied the same Bayesian analysis to the *Distractor-Fixated trials*. The Bayesian analysis revealed that the data are 2.5 * 10^3^ times more likely under H0 than H1: that is, on *Distractor-Fixated trials*, there was no effect of Prompt Type on the probability of observing double-checks (*Unfamiliar*/*Negative*: *M* = 2%, *SD* = 10%; *Familiar*/*Positive*: *M* = 4%, *SD* = 17%). The data were 70 times more likely under H1 than H2. There were no interactions – the three tasks yielded a similar pattern of results (see Figure 2).

### Toddlers’ Reaction Times to Point Analysis

If rejection of the competitor (along with disjunctive commitment that at least one of the two options is correct) is driving toddlers’ confidence in choosing the target object on *Unfamiliar*/*Negative trials*, then we would expect toddlers’ reaction times to speed up after having carried out the double-check that signals the explicit rejection of the competitor. Specifically, we considered those trials where the preschooler’s final look is to the target (n = 365) and asked how long they wait until pointing after they have reoriented their gaze to that object, as an index of their confidence that the object they are fixating is the target of the experimenter’s prompt.

We fit mixed effects models predicting reaction times to point (i.e., time of pointing to the target minus the time from the beginning of the final look to the target) across the 3 Tasks and 4 Trial Types: *Familiar*/*Positive trials* (n = 204) encompassing all types of fixation patterns; *Novel*/*Negative trials*, *Target-Fixated*, *With-No-Double-Check* (n = 33) where participants fixated the target at the time of the prompt but the single-double-check-then-point pattern was not performed; *Novel*/*Negative Trials*, *Distractor-Fixated* (n = 75) where participants fixated the competitor at the time of the prompt; and *Novel*/*Negative Trials*, *Target-Fixated*, *With-Double-Check* (n = 53) where participants fixated the target at the time of the prompt and the single-double-check-then-point pattern was performed.

Conservatively, we measure reaction times only from the beginning of the last fixation on the target object regardless of trial type. Each model had a fixed effect for Task and a random intercept for participant. The first model “H0” had no additional effects. The second model “H1” had a fixed effect for Trial Type (i.e., *Familiar*/*Positive* vs. *Unfamiliar*/*Negative*). The third model “H2” had a fixed effect for Trial Type and an interaction effect between Trial Type and Task. The data supported the model with only the main effects of Trial Type and Experiment over the other two (each BF > 10 * 3). Hence, we followed with planned pairwise comparisons between each *Novel*/*Negative trial type* and the *Familiar* (*Positive*) *trials*, which we use as a benchmark of children’s reaction time when they had positive knowledge of the prompt target.

**Figure 3.**
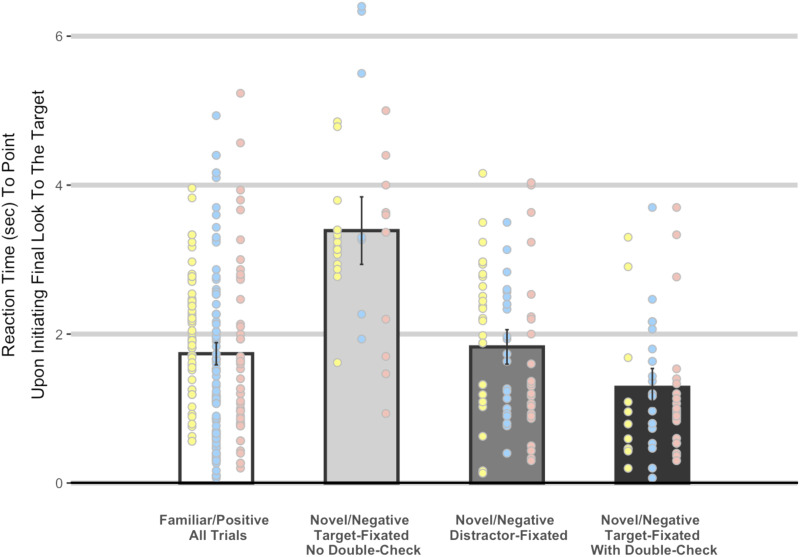
Reaction times to point at the target as a function of trial type. Bars reflect the reaction time to point to the target object minus the time at which the toddler has initiated their final fixation of the target object—that is, once you have your eyes on the target, how certain are you that it is the correct choice as measured by how long do you delay before pointing. Each dot is an individual trial from one of our three tasks (Yellow = Word Learning; Blue = Social Learning; Pink = Explicit Negation). Bars represent the universal mean across all three tasks, and error bars represent the 95% Confidence Interval across all observations.

First, we found that reaction times on *Novel*/*Negative trials*, *Target-Fixated*, *With-No-Double-Check* (*M* = 3358 ms, *SD* = 1400 ms) were significantly longer than those on the *Familiar*/*Positive trials* (*M* = 1787 ms, *SD* = 610 ms; *p* < .001). The slower reaction times in *Novel*/*Negative trials*, *Target-Fixated*, *With-No-Double-Check* are consistent with preschoolers performing disjunctive inference in their memory without shifting their gaze, a process that takes time, and thus, the reaction times are longer. Second, we found no difference in toddlers’ reaction times on *Novel*/*Negative Trials*, *Distractor-Fixated* (*M* = 1809 ms, *SD* = 923 ms) and those on *Familiar*/*Positive trials* (*p* < .959). This is consistent with the proposal that, once children have rejected the distractor object, they achieve high certainty to point at the target object, comparable to the *Familiar*/*Positive trials*. Lastly, toddlers were *faster* to point to the target object on *Novel*/*Negative Trials*, *Target-Fixated*, *With-Double-Check* (*M* = 1365 ms, *SD* = 928 ms) than on *Familiar*/*Positive trials* (*p* < .05). This is also consistent with the proposal that, once children have rejected the distractor object, they achieve high certainty to point at the target object, comparable to, or even faster than, the *Familiar*/*Positive trials*. These reaction times can be seen in Figure 3.

### Toddlers’ Redundant Saccades Analysis

If toddlers were indeed performing Disjunctive Inference on *Unfamiliar*/*Negative trials*, then they should (ideally) be confident in their decision. That is, if toddlers construe each trial as a 2-alternative forced-choice, having moved their attention to the distractor object and rejected it as a possible target, toddlers can move directly to the target and choose it, without further inspection of the two objects. One form of data that might index the level of toddlers’ confidence post-elimination of the alternative is children’s probability of making *redundant eye movements* between the objects before pointing – after having had the opportunity to check the distractor and move to the target. We predicted that, if toddlers work through Disjunctive Inference and have high-confidence in their decision, redundant saccades would *not* be higher in the *Unfamiliar*/*Negative* condition than in the *Familiar*/*Positive* condition. Furthermore, if toddlers use the same inference in all of the three tasks, we expect no interaction effect between tasks and prompt types.

To test these predictions, we scored the presence of redundant saccades in a trial if, during the decision phase, a participant performed one or more saccades between the two potential targets above those predicted by our account (see Figure 1).

We compared the probability of redundant saccades in the *Distractor-Fixated Familiar*/*Positive trials* with the *Unfamiliar*/*Negative* ones, with the same Bayesian analysis used for the double-check. By our hypothesis, there should be no difference in the probability of redundant saccades in the two conditions. Indeed, the analysis revealed that the data are 7.7 * 10^2^ times more likely under H0 than H1: in the *Distractor-Fixated trials*, there is no effect of Prompt Type on the probability of redundant saccades (*Unfamiliar*/*Negative*: *M* = 7%, *SD* = 17%; *Familiar*/*Positive*: *M* = 7%, *SD* = 21%). Furthermore, the analysis revealed that the data are 1.5 * 10^2^ times more likely under H1 than H2: there is no interaction, and the three tasks resulted in a similar pattern of results. This argues that rejection of the competitor object increases the certainty that the participant has that the target object is the correct target – up to a level equivalent to the Familiar/Assertion prompts.

We also compared the probability of redundant saccades in the *Target-Fixated trials* by the same analysis. The analysis revealed that the data are 4.9 * 10^3^ times more likely under H1 than H0: in the Target Fixated trials, redundant saccades are *more likely* in the *Familiar*/*Positive* condition than in the *Unfamiliar*/*Negative* one (*Unfamiliar*/*Negative*: *M* = 2%, *SD* = 10%; *Familiar*/*Positive*: *M* = 29%, *SD* = 32%). Furthermore, the analysis revealed that the data are 1.5 * 10^2^ times more likely under H1 than H2: there is no interactions and the three tasks resulted in a similar pattern of results (see Supplementary Figure 1).

## DISCUSSION

Toddlers robustly succeeded in inferring the name of a novel object, the preference of a novel character and the winner of a race based on explicit verbal negation, in line with previous findings that even infants can find the referent of a novel name (e.g., Halberda, 2003; Pomiechowska et al., 2021), and that toddlers respond to verbal negation (Austin et al., 2014; Feiman et al., 2017; Grigoroglou et al., 2019). It was however unknown whether, in doing so, they deployed a logical inference (Mody & Carey, 2016, see also Leahy & Carey, 2020), as adults do (Halberda, 2006).

Toddlers’ spontaneous eye-movements in the three experiments revealed a positive answer to our query. For all three tasks, on the critical *Target-Fixated Unfamiliar*/*Negative trials*, children were significantly more likely to visually double-check the competitor before choosing the target object (e.g., novel object), compared to trials when they were fixating the target (e.g., ball) but hearing a *Familiar*/*Positive prompt* (where Disjunctive Inference is unnecessary; Figure 2). This behavioral pattern supports the inference that toddlers performed Disjunctive Inference on *Unfamiliar*/*Negative trials*. Toddlers also performed few double-checks on trials where they were fixating the distractor at the time of the prompt, demonstrating that when double-checks are unnecessary (because children are already fixating the to-be-rejected distractor), children do not tend to perform them—that is *Unfamilaliar*/*Negative prompts* do not lead to needless increases in gaze shifts.

What about the limitations of the present and previous research? When considering only success or failure, without considering the looking pattern that leads to success, 2-alternative forced-choice tasks where participants are prompted to discount one of the alternatives are inconclusive regarding the logical character of the correct response. After hearing an unfamiliar name/preference, an illogical participant could choose the unfamiliar option by simply avoiding the familiar one, without ever updating (increasing) the expectation that the unfamiliar option may be the target of the unfamiliar prompt (Mody & Carey, 2016; Watson et al., 2001) or by being positively motivated to “map-novelty-to-novelty”. These non-logical accounts present a challenge to the forced choice paradigm across modalities (visual and auditory, Call, 2004; verbal negation, Feiman et al., 2017) and task domains (objects and causality, Feiman et al., 2022). Even doubling the pairs of alternatives (Ferrigno et al., 2021; Mody & Carey, 2016), does not fully counter this deflationary account, since participants could avoid one cup and pick the other focusing on only one pair of alternatives at a time (Engelmann et al., 2023; Gautam et al., 2021).

In our experiment, we tested the presence/absence of logical updates by comparing the rate of redundant (saccades) performed by the toddlers between Familiar and Unfamiliar trials. One previous study has measured redundant checks before making a choice as an indicator of a logical update or lack thereof for apes and toddlers (Call & Carpenter, 2001). Here, we found that the probability of performing redundant saccades in the *Distractor-Fixated Unfamiliar*/*Negative trials* was as low as in the *Distractor-Fixated Familiar*/*Positive trials*, which is when participants have a conclusive expectation about the target of the prompt. Therefore, as mentioned earlier, hearing *Unfamiliar*/*Negative prompts* does not simply lead children to perform redundant and inconclusive gaze shifts but rather to a systematic and efficient scanning pattern leading to a firm expectation.

A possible reply to the redundant saccades analysis is that participants in the *Unfamiliar*/*Negative trials* might have refrained from redundant saccades and pointed at the correct object even if they remained uncertain about it because they were certain that the competitor object was not the target. To address this proposal^3^ we compared participants’ reaction times to point (i.e., time of pointing to the target minus the time from the beginning of the final look to the target) in the *Familiar*/*Positive* and the *Novel*/*Negative trials*. We found that in the *Target-Fixated Novel*/*Negative trials* participants who didn’t perform the double-check had slower reaction times than in the *Familiar*/*Positive* trials, suggesting that they needed the time to mentally exclude the competitor. In contrast, participants in the *Target-Fixated Novel*/*Negative trials* who did perform the double-check were faster than in the *Familiar*/*Positive trials*. This result shows that, after excluding the competitor, children’s certainty about the target in the *Novel*/*Negative trials* was comparable, or superior, to that in the *Familiar*/*Positive trials*.

A further concern is the status of the verbal negation “Not” in the Explicit Negation task. Even if infants understand “Not” as expressing a logical operation, there are multiple ways to use negation to choose the correct option in our task. A first strategy is to use Disjunctive Inference (i.e., is the winner the lion on the left OR the blue jay on the right? The winner is not the lion. Therefore, the winner is the blue jay). In this case, the children appropriately understand the negation to be negating the possibility that the lion is the winner, *it is not the case that the lion is the winner*. A second strategy is to interpret the negation, not sententially, but to form a contradictory concept like *the winner is [a non-lion]*. Searching for a non-lion is sufficient to “positively” select the blue-jay without the requirement to consider and exclude the possibility that the lion is the winner.^4^ However, the similarities in reaction time to point across the three tasks for the various trial types (Figure 3) suggests that toddlers’ relied on a common strategy across the three tasks.

Crucially, the contradictory concept strategy makes a similar prediction to the novelty-to-novelty account. If toddlers use the contradictory concept strategy, they do not need to exclude the competitor, and therefore, an increase in the probability of the double-check is not expected. Against the predictions of this contradictory concept strategy, we found that toddlers were more likely to double-check the competitor in the negative condition of the Explicit Negation task. This suggests that, like adults and preschoolers in similar tasks (Halberda, 2006), toddlers are using Disjunctive Inference to apply the negative prompt to solve the task: that is, they respond to the negative prompt by considering and excluding the competitor.

Finally, it has been proposed that children might map-novel-names-onto-novel-objects not by considering an exhaustive list of alternatives but by simply applying the one-to-one mapping principle that “each object has at most one label” (Feiman et al., 2022). In evaluating this proposal, it is important to recall that from the principle “each object has at most one label” and “the familiar object has the label “Ball”” it does not follow that ““dax” refers to the unfamiliar object”, since, e.g., “dax” might refer to neither of them. For the mapping to follow, one needs the further assumption that ““dax” must refer to one of the two objects and not both”, i.e., a representation of which objects form an *exhaustive* list of potential referents. Hence, toddlers’ mapping of novel names to novel objects, when driven by the elimination of the competitor, is best explained by Disjunctive Inference. Nevertheless, future research can further explore the relationship between the one-to-one mapping principle and the preverbal Disjunctive Inference and ask whether the latter can also emerge in situations when the one-to-one mapping doesn’t apply (e.g. when multiple non-mutually exclusive mappings are possible).

Thus, across tasks and trials, toddlers’ looking behavior was consistent with them making Disjunctive Inference on precisely those trials where it would be helpful. The predictions generated by hypothesizing continuity in logical development were born out, indicating that, before robust evidence of logical vocabulary use for “or” (Chierchia et al., 2001; Mody & Carey, 2016; Morris, 2008; Singh et al., 2016), toddlers used Disjunctive Inference in each of our three tasks, as adults and older children do.

Our findings show that toddlers can represent two alternatives in disjunctive relation (e.g., A OR B) and then infer the correct one by excluding the other (e.g., A is ruled out; THEREFORE, B). Might toddlers’ responses in each of our three tasks emerge from *a single*, *domain-specific competence*? This is unlikely since, to answer correctly in each experiment, they needed to rely on highly heterogeneous kinds of competencies: excluding that an unknown name refers to a known object, excluding that a puppet holds potentially conflicting preferences, and understanding that the word “not” was used to deny the victory of one of the competitors in a race.

Might our three tasks draw instead upon *three different*, *task-specific understandings of Disjunctive Inference*? Across the three tasks, we observed a similar increase in the probability of a single double-check when Disjunctive Inference was required to respond to an unfamiliar/negative prompt, while the probability of redundant saccades, above those predicted by the process of elimination, was not higher than in response to familiar/positive information. This pattern strongly suggests that the same inferential strategy is deployed across the three distinct task domains. Moreover, it seems unparsimonious to assume that toddlers have domain-specific cognitive systems that support generalizations such as “*in a game, at most one of the players win”* (Experiment 3) and “*every animal has only one preference”* (Experiment 2). Toddlers’ inferences in these tasks seem to reflect ad-hoc assumptions that they form in the experiment (e.g., the lion OR the bluejay will win the race) rather than the application of their knowledge of specific domains.

Hence, toddlers’ success is best explained by a general capacity to recognize ad-hoc logical dependencies – rather than domain-specific knowledge. In summary, the success we observed in the race task and in the other two entirely unrelated content domains, and the strikingly similar pattern of children’s eye movements across all of these, suggests that the toddlers reasoned in the same way across very different contents. These early emerging domain-general logical inferences may reflect the foundation of general-purpose learning, planning, and decision-making.

Our results point to important questions for future research: do infants and toddlers use the same general logical operation to discount alternatives when they reason disjunctively in different domains? And is the mastery of verbal negation a prerequisite for this abstract form of elimination? While our experiments support a domain-general form of Disjunctive Inference, they are compatible with both domain-general notions of elimination of an alternative (*““dax” doesn’t refer to the familiar object”* rules out *““dax” refers to the familiar object”*), and domain-specific ones (e.g., *““dax” is a novel name”* rules out *““dax” refers to the familiar object”*). In other words, domain-general negation is not required for general forms of Disjunctive Inference.^5^ Future research is required to ask whether when infants and toddlers reason disjunctively in different domains, they are using the same mental operator of negation (Feiman et al., 2022) or a variety of domain-specific contrary concepts (e.g., empty/full; familiar/unfamiliar).

A follow-up to our between-subjects approach would be to investigate if there are stable individual differences in the type of inferential capacities studied here. With such an approach, one might expect to find correlations in performance across the various tasks that may employ a logical inference. However, one caution for this approach is that if children are prolific at using this strategy across contexts, there might not exist stable individual differences in performance (e.g., all children might do well)—such as in the three overwhelming successes seen here. In such a situation (or, similarly, other situations where an ability is fundamental and observed early), one might not find correlations in performance across tasks even though the underlying ability is, in fact, shared and domain-general. For this reason, we recommend that such a within-subjects approach should focus either on younger children than were tested here or on a different task with higher logical demands in order to increase variance.

Further research will be indispensable for uncovering the origins of the broad class of logical abilities that help get thinking off the ground (Chierchia, 2013; Icard & Moss, 2014; Knowlton et al., 2022; Pietroski, 2018). Experience, maturation, and the acquisition of the logical language may all play critical roles in empowering adult-like logical cognition. Here, we uncovered strong evidence of continuity in the development of Disjunctive Inference: the ability to reason via at least one domain-general logical computation appears to be in place by the third year of life.

## ACKNOWLEDGMENTS

We thank the parents and children participating in this study. We are grateful to L. Feigenson, B. Landau, L. L. Bonatti, J. Trueswell, Z. Ovans, S. Carey, R. Feiman, B. Leahy and two anonymous reviewers for their very valuable comments. We thank S. Carey – who financially and intellectually supported this research. We gratefully acknowledge J. Bowen for statistics consulting and help in constructing Figure 3.

## FUNDING INFORMATION

This research was supported by funds for a postdoctoral fellowship from the James S. McDonnell Foundation.

## AUTHOR CONTRIBUTIONS

J.H. designed the study; J.H. performed the research; J.H. and N.C.-A., analyzed the data; and J.H. and N.C.-A., wrote the paper.

## DATA AVAILABILITY

All data and analysis code are available at: https://osf.io/ge8zh/?view_only=b00a1c172cea4ae9aa3be085d29237f3.

## Notes

^1^ We adopt the term “Disjunctive Inference” to refer to a broader type of inference of which Disjunctive Syllogism is a special case. In Disjunctive Inference, two (or more) alternatives are represented in a disjunctive relation, entailing that at least one of them must be true, so evidence against one alternative supports the other(-s). The *disjunctive relation* can be represented with an operator of disjunction (“The ball is inside the cup OR the toy car is inside the cup”; Braine & O’Brien, 1998) or with a different format of representation, like, for example, an exhaustive list of mutually exclusive models, or simulations, of alternative possibilities ({“the ball is inside the cup”, “the toy car is inside the cup”}; Johnson-Laird, 2010). The *elimination of one alternative* can be performed in several ways, for example, via mutually-exclusive representations (e.g., “ the ball is outside the cup” rules out “the ball is inside the cup”), or via the operator of negation (e.g., “ the ball is NOT in the cup” rules out “the ball is in the cup” (see Feiman et al., 2022, for a helpful discussion of a related distinction). Hence, Disjunctive Syllogism is a special case of Disjunctive Inference, where the disjunctive relation is represented with an operator of disjunction, and the alternative elimination is attained with an operator of negation. It is an open empirical question whether infants and children use Disjunctive Syllogism or another format of representation when they reason disjunctively (see Cesana-Arlotti, 2023).^2^ Our predictions are conservative because we assume that participants will draw the conclusion of Disjunctive Inference and choose an option as soon as they mentally exclude one alternative, which leaves time for a single double-check.^3^ We thank an anonymous reviewer for proposing it.^4^ We thank an anonymous reviewer for suggesting to consider this alternative logical strategy.^5^ Domain-general disjunctive inference is consistent with a domain-specific exclusion of a disjunct (e.g., cup A has the prize OR in cup B has the prize; cup A is excluded because it is empty; therefore, cup B has the prize). In this case, the *positive*, implicitly negative domain-specific notion of *empty* drives the general exclusion of the empty cup thereby increasing the certainty that the target cup is the correct option. It is really this general update of one expectation by the exclusion of an alternative and not the specific basis for it that we are testing here with the double-checks.

## Supplementary Material


