# Supplementary material for: A Continuity in Logical Development: Domain-General Disjunctive Inference by Toddlers
Source: Open Mind (Camb). 2024 Jun 28;8:809–25. doi: 10.1162/opmi_a_00148 (PMC11226237; doi:10.1162/opmi_a_00148)
Supplement: Supplementary file 1 [file opmi-08-809-s001.docx]

**Supplementary Information For**

**A continuity in logical development: domain-general disjunctive inference by toddlers**

Authors: Nicolò Cesana-Arlotti^1*^, Justin Halberda^2*^

^1^ Department of Psychology, Yale University, New Haven, CT, USA

^2^ Department of Psychological and Brain Sciences, Johns Hopkins University, Baltimore, MD, USA

Correspondence to: nicolo.cesana-arlotti@yale.edu, halberda@jhu.edu

**This PDF file includes:**

Supplementary Note 1: supporting methods

Supplementary Note 2: exploratory analysis of the pointing response with excluded participants

Supplementary Note 3: double-check and redundant saccades analysis details with Tables S1 to S5

Supplementary Note 4: exploratory analyses of the double-check and redundant saccades with excluded participants.

Supplementary Note 5: exploratory analysis of the double-check probability with all trial types and compared at once.

Supplementary Figure 1

Supplementary References

**Supplementary Note 1: supporting methods**

In the *Word Learning task,* children were given four warm-up trials. The experimenter placed two familiar objects simultaneously on the table (e.g., a brush, cup), then ducked his head behind the screen and asked “Can you point at the ­____ [e.g., brush]?”. The trial ended when children pointed to or grasped the requested object. If children did not respond, the experimenter raised his head and repeated the question. Next, children were presented with 12 test trials. These were structured like warm-up trials, except they could come in three types. In Familiar-Name trials, children either saw two familiar objects (4 trials) or one familiar object and one unfamiliar object (4 trials), and the experimenter asked them to point to a familiar object using its familiar name. In these trials, children could use their knowledge of the name’s meaning to directly identify the target object. In Unfamiliar-Name trials (4 trials), children saw one familiar and one unfamiliar object, and the experimenter asked them to point at the unfamiliar object using an unfamiliar name. On these trials, the child did not know the referent of the unfamiliar name but could identify it by reasoning disjunctively (e.g., “dax” must refer to one object OR the other; “dax” does not refer to the ball; THEREFORE, “dax” must refer to the unfamiliar object). No object name was ever repeated (whether as warm-up or test), and no more than two consecutive trials were of the same type. The side of correct response varied pseudo-randomly.

In the *Social Learning task*, children were introduced to three stuffed animals (e.g., Elephant, Frog, Tiger) and their distinctive preferences. The experimenter began by introducing Elephant in the first-person (e.g., "Hi, I'm Elephant, nice to meet you!"). Next, Elephant told children something that he liked-- either a food or an activity (e.g., "Do you know what I like? I like soccer!"). This process of introducing the animal and its food or activity preference was repeated for Frog. Next the experimenter brought out Frog and Elephant simultaneously, then ducked his head behind the screen and stated aloud either Elephant or Frog’s preference (e.g., "I like soccer!"). He then picked his head up from behind the screen and asked children, "Who said that?" If children responded correctly, the experimenter said, e.g., "That's right! Elephant does like soccer!" If children responded incorrectly, the experimenter said, e.g., "No, Frog doesn't like soccer; Elephant likes soccer!" Next, the experimenter brought out a third stuffed animal who told children what they liked. These original three *Familiar Animals* were used throughout the testing session-- at least one Familiar Animal was used on each subsequent trial. The experimenter repeated practice trials like the one described above using different combinations of the Familiar Animals, keeping his head behind the screen, until it was determined that children had successfully learned what each of them liked (as defined by getting at least four consecutive trials correct; *M* = 8.4 trials to reach criterion; range: 5 to 14 trials). After reaching criterion, each child was presented with 14 test trials. These were structured like the practice trials, except they could come in three types. In Familiar-Preference trials children either saw two Familiar Animals, or one Familiar Animal and one Unfamiliar Animal, and the experimenter stated an animal’s familiar preference (e.g., “I like soccer!”) and asked children which animal had said that. On these trials, children could use their knowledge of the preference-puppet mapping (i.e., the familiar puppet’s distinctive preference) to identify who was speaking. In Unfamiliar-Preference trials (at least three trials) children saw one Familiar and one Unfamiliar animal, the experimenter stated an animal’s unfamiliar preference (e.g., “I like taking walks!”), and children were asked which animal had said that. On these trials, children did not know the owner of the unfamiliar preference and had to identify it by reasoning disjunctively (e.g., EITHER Elephant OR Bear likes pizza; but Elephant likes soccer, not pizza (assuming each puppet has exactly one distinctive preference); THEREFORE, the Bear must like pizza. No Unfamiliar Animal was presented more than once, no more than three consecutive trials were of the same type, and the side of correct response varied pseudo-randomly.

In the *Explicit Negation task*, children were introduced to two stuffed animals (e.g., Frog, Tiger), and the experimenter explained that they were going to race to reach a finish line (a line of colored tape, near the child, on the table), but that children needed to help by making the winning animal cross the finish line. The experimenter then ducked his head behind the screen, said, “ready, GO!” and started to move the animals toward the finish line (i.e., towards the child). Before they crossed it, paused and said, “And the winner is the ____ [e.g., Tiger]”, and then said, “Can you help me make the winner finish the race?” (this explicit request was made only on the first trial, which was considered a practice trial). The trial ended when children pointed at or grasped one of the animals. Practice trials were repeated using different combinations of stuffed animals until it was determined that children understood the game (*M* = 2.2 trials; range: 1-4 trials). Test trials were structured like practice trials, except they could come in two types. On Assertion trials the experimenter indicated the winner by saying “And the winner is the ___ [e.g., Elephant]!” On these trials, children could directly identify the winner because its identity was explicitly stated by the experimenter. On Negation trials the experimenter indicated the winner by saying “And the winner is NOT the ____ [e.g., Lion]!” On these trials, children could identify the winner reasoning disjunctively (i.e., EITHER Elephant OR Lion will win the race. Lion is NOT the winner; THEREFORE, Elephant must be the winner). Children were presented with up to 12 test trials, depending on their interest in the task as judged by the experimenter (*M* = 9.25 trials; range: 7-12 trials). Half were Negation trials and half Assertion trials. No more than two consecutive trials were of the same type (Assertion vs. Negation) and the side of the correct response varied pseudo-randomly.

**Supplementary Note 2: exploratory analysis of the pointing response with excluded participants**

To probe the robustness of toddlers’ success in inferring the target of the experimenter prompt, we also ran an exploratory analysis including the seven participants that were excluded from the analysis since they lacked valid trials in at least one of the four conditions. Sign tests found that children remained near ceiling on all trial types across the three tasks: Word Learning, *M_Unfamiliar-Name_* = 87% correct (n = 16, *p* < .001), *M_Familiar-Name_* = 97% (n = 16, *p* < .001); Social Learning, *M_Unfamiliar-Preference_* = 96% (n = 14, *p* < .001), *M_Familiar-Preference_* = 96% (n = 14, *p* < .001); Explicit Negation, *M_Negation_* = 91% (n = 13, *p* < .001), *M_Assertion_* = 98%, (n = 13, *p* < .001).

**Supplementary Note 3: double-check and redundant saccades analysis details**

To test our hypothesis that toddlers deployed a Disjunctive Inference to find the target of the *Unfamiliar/Negation prompts*, we compared our predictions about toddlers’ visual scanning strategies (see Main Text Figure 1) with their eye movements (see Table S1)

To test the predictions that the probability that a participant will execute a single double-check before pointing is higher in the *Unfamiliar/Negation Target-Fixated trials*, we fit mixed-effects logistic regression models the presence of exactly one double-check (using the lm4 package for R, see supplementary code). Each model had a fixed effect for Task (i.e., Word-Learning vs. Social-Learning vs. Explicit-Negation) and a random intercept for Participant, nested in Task. The "null-hypothesis" Model**_0_** *Double-Check ~ Task + (1| Participant)* had no effect for Prompt Type (i.e., *Familiar/Assertion* vs. *Unfamiliar/Negation*). The “MainEffects” Model**_1_** *Double-Check ~ Task + PromptType + (PromptType| Participant)* had the effect for Prompt Type. The "interaction" Model**_2_** *Double-Check ~ Task + PromptType + PromptType:Task + (PromptType| Participant)* also included the interaction between Prompt Type and Task. Tables S2 and S3 show the fixed parameter estimates for each model yielded for the data of the *Target-Fixated trials* and *Distractor-Fixated trials*, respectively.

We used Bayesian information criteria (BIC; (Schwarz, 1978)) to guide model selection. BIC is a measure of goodness-of-fit, balancing the fit of the candidate model to the data with model complexity by including a penalty for the number of estimated parameters. Generally, the model with the lowest BIC is considered to be the best. However, to quantify the evidence in support of one model over another, BIC-derived Bayes factors can be computed (Wagenmakers, 2007). In the *Target Fixated trials*, the null-hypothesis Model**_0_** scored BIC = 270.79, the main effect Model**_1_** scored BIC = 262.21, and the interaction Model**_2_** scored BIC = 272.22. A comparison between the null-hypothesis Model**_0_** and the main effect Model**_1_** yielded a Bayes factor BF10 = 73, and a comparison between the main effect Model**_1_** and the interaction Model**_2_** yielded a Bayes factor BF12 = 149, indicating strong evidence for the main effect model (Jeffreys, 1985). In the *Distractor Fixated trials*, the null-hypothesis model scored BIC = 62.91, the main effect model scored BIC = 78.56, and the interaction model scored BIC = 87.06. A comparison between the null-hypothesis Model**_0_** and the main effect Model**_1_** yielded a Bayes factor BF01 = 2496, and a comparison between the main effect Model**_1_** and the interaction Model**_2_** yielded a Bayes factor BF12 = 70, indicating strong evidence for the null-hypothesis model. In summary, our analysis shows that in the *Target Fixated trials*, toddlers' were *more* likely to perform a single double-check before pointing in response to an *Unfamiliar/Negation* *prompt* than to a *Familiar/Assertion prompt*. In contrast, in the *Distractor Fixated trials,* the double-check probability was not modulated by the prompt type. These results confirm the prediction made by the Disjunctive Inference.

| **Eye-movements Summary** | | | | | |
| --- | --- | --- | --- | --- | --- |
| Task | Object Fixated | Prompt Type | Mean Gaze Shifts N | Double Check Frequency | Redundant Saccades Frequency |
| Word Learning | Target-Fixated | Familiar/Positive | 0.2 | 7% | 10% |
| Word Learning | Target-Fixated | Unfamiliar/Negative | 0.8 | 40% | 0% |
| Word Learning | Distractor-Fixated | Familiar/Positive | 1 | 8% | 8% |
| Word Learning | Distractor-Fixated | Unfamiliar/Negative | 1.1 | 7% | 10% |
| Social Learning | Target-Fixated | Familiar/Positive | 1.1 | 33% | 50% |
| Social Learning | Target-Fixated | Unfamiliar/Negative | 1.5 | 69% | 4% |
| Social Learning | Distractor-Fixated | Familiar/Positive | 1 | 3% | 6% |
| Social Learning | Distractor-Fixated | Unfamiliar/Negative | 1 | 0% | 4% |
| Explicit Negation | Target-Fixated | Familiar/Positive | 0.5 | 28% | 28% |
| Explicit Negation | Target-Fixated | Unfamiliar/Negative | 1.8 | 62% | 3% |
| Explicit Negation | Distractor-Fixated | Familiar/Positive | 1.1 | 0% | 6% |
| Explicit Negation | Distractor-Fixated | Unfamiliar/Negative | 1.1 | 0% | 7% |

**Table S1.** **Summary of participants' eye movements by condition.** The average number of gaze shifts between the object, frequency of the single double-check, and frequency of at least one redundant saccade.

|  |  |  |  |  |
| --- | --- | --- | --- | --- |
| **Null-hypothesis Model_0_: Double-Check ~ Task + (1\| Participant)** | | | | |
|  | Estimate | *SE* | *z* | *p* |
| (Intercept) | -1.2528 | 0.3030 | -4.134 | <.0001* |
| Social Learning | 1.2268 | 0.3792 | 3.235 | <.01* |
| Explicit Negation | 1.2891 | 0.4057 | 3.178 | <.01* |
|  |  |  |  |  |
| **Main Effect Model_1_: Double-Check ~ Task + PromptType + (PromptType\| Participant)** | | | | |
|  | Estimate | *SE* | *z* | *p* |
| (Intercept) | -2.0973 | 0.3852 | -5.445 | <.0001* |
| Unfamiliar/Negative | 1.5771 | 0.3343 | 4.718 | <.0001* |
| Social Learning | 1.4704 | 0.4139 | 3.552 | <.001* |
| Explicit Negation | 1.2083 | 0.4326 | 2.793 | <.01* |

| **Interaction Model_2_: Double-Check ~ Task + PromptType + PromptType:Task + (PromptType\| Participant)** | | | | |
| --- | --- | --- | --- | --- |
|  | Estimate | *SE* | *z* | *p* |
| (Intercept) | -2.3979 | 0.6030 | -3.976 | <.0001* |
| Unfamiliar/Negative | 2.0232 | 0.7191 | 2.814 | <.01* |
| Social Learning | 1.8299 | 0.6751 | 2.710 | <.01* |
| Explicit Negation | 1.5712 | 0.7543 | 2.083 | <.05* |
| Unfamiliar/Negative: Social Learning | -0.6079 | 0.8763 | -0.694 | .48786 |
| Unfamiliar/Negative: Explicit Negation | -0.5499 | 0.9279 | -0.593 | .55342 |

**Table S2.** **Model_0_ , Model_1_ and Model_2_ for the *Target Fixated trials****.* Estimated (fixed) regression coefficients with their standard errors, *z*- and *p*-values (* *p* < .05).

|  |  |  |  |  |
| --- | --- | --- | --- | --- |
| **Null-hypothesis Model_0_: Double-Check ~ Task + (PromptType\| Participant)** | | | | |
|  | Estimate | *SE* | *z* | *p* |
| (Intercept) | -2.9087 | 0.5929 | -4.906 | <.0001* |
| Social Learning | -0.6886 | 0.9302 | -0.740 | .459 |
| Explicit Negation | -19.6573 | 6748.8098 | -0.003 | .998 |
|  |  |  |  |  |
| **Main Effect Model_1_: Double-Check ~ Task + PromptType + (PromptType\| Participant)** | | | | |
|  | Estimate | *SE* | *z* | *p* |
| (Intercept) | -2.91577 | 0.74081 | -3.936 | <.0001* |
| Unfamiliar/Negative | 0.01508 | 0.94729 | 0.016 | .987 |
| Social Learning | -0.68599 | 0.94442 | -0.726 | .468 |
| Explicit Negation | -19.64651 | 6710.07951 | -0.003 | .998 |

| **Interaction Model_2_: Double-Check ~ Task + PromptType + PromptType:Task + (PromptType\| Participant)** | | | | |
| --- | --- | --- | --- | --- |
|  | Estimate | *SE* | *z* | *p* |
| (Intercept) | -3.4012 | 1.0165 | -3.346 | <.001* |
| Unfamiliar/Negative | 0.8755 | 1.2543 | 0.698 | 0.4852 |
| Social Learning | 0.1625 | 1.2462 | 0.130 | 0.8962 |
| Explicit Negation | -19.1649 | 9639.2285 | -0.002 | 0.9984 |
| Unfamiliar/Negative: Social Learning | -20.1273 | 9894.2948 | -0.002 | 0.9983 |
| Unfamiliar/Negative: Explicit Negation | -0.8755 | 13500.215 | 0.000 | 0.9999 |

**Table S3.** **Model_0_ , Model_1_ and Model_2_ for the *Distractor Fixated trials****.* Estimated (fixed) regression coefficients with their standard errors, *z*- and *p*-values (* *p* < .05).

The same model selection procedure was used to test the prediction that participants' execution of redundant saccades is not more probable in the *Unfamiliar/Negation trials.* Tables S4 and S5 show the fixed parameter estimates for each model yielded for the data of the *Target-Fixated trials* and *Distractor-Fixated trials*, respectively. In the *Target Fixated trials*, the null-hypothesis Model**_0_** scored BIC = 190.41, the main effect Model**_1_** scored BIC = 173.41, and the interaction Model**_2_** scored BIC = 183.47. A comparison between the null-hypothesis Model**_0_** and the main effect Model**_1_** yielded a Bayes factor BF10 = 4908, and a comparison between the main effect Model**_1_** and the interaction Model**_2_** yielded a Bayes factor BF12 = 152, indicating strong evidence for the main effect model. In the *Distractor Fixated trials*, the null-hypothesis model scored BIC = 108.64, the main effect model scored BIC = 121.94, and the interaction model scored BIC = 131.91. A comparison between the null-hypothesis Model**_0_** and the main effect Model**_1_** yielded a Bayes factor BF01 = 771, and a comparison between the main effect Model**_1_** and the interaction Model**_2_** yielded a Bayes factor BF112 = 146, indicating strong evidence for the null-hypothesis model. In summary, our analysis shows that in the *Target Fixated trials*, toddlers were *less* likely to perform redundant saccades before pointing in response to an *Unfamiliar/Negation* *prompt* than to a *Familiar/Assertion prompt*. Crucially, in the *Distractor Fixated trials,* the prompt type did not modulate the probability of redundant saccades. These results indicate that the confidence of participants' decisions was not lower in the *Unfamiliar/Negation* *trials* than in the *Familiar/Assertion trials.*

|  |  |  |  |  |
| --- | --- | --- | --- | --- |
| **Null-hypothesis Model_0_: RedundantSaccades ~ Task + (1\| Participant)** | | | | |
|  | Estimate | *SE* | *z* | *p* |
| (Intercept) | -2.6912 | 0.5167 | -5.209 | <.0001* |
| Social Learning | 1.8378 | 0.5735 | 3.204 | <.01* |
| Explicit Negation | 0.9205 | 0.6428 | 1.432 | .1521 |
|  |  |  |  |  |
| **Main Effect Model_1_: RedundantSaccades ~ Task + PromptType + (PromptType\| Participant)** | | | | |
|  | Estimate | *SE* | *z* | *p* |
| (Intercept) | -2.1228 | 0.5291 | -4.012 | <.0001* |
| Unfamiliar/Negative | -3.0624 | 0.7591 | -4.034 | <.0001* |
| Social Learning | 1.9813 | 0.5986 | 3.310 | <.001* |
| Explicit Negation | 1.3610 | 0.6776 | 2.009 | <.05* |

| **Interaction Model_2_: RedundantSaccades ~ Task + PromptType + PromptType:Task + (PromptType\| Participant)** | | | | |
| --- | --- | --- | --- | --- |
|  | Estimate | *SE* | *z* | *p* |
| (Intercept) | -2.0794 | 0.5303 | -3.921 | <.0001* |
| Prompt Type | -18.3813 | 3237.1461 | -0.006 | .9954 |
| Social Learning | 1.9516 | 0.6056 | 3.223 | <.001* |
| Explicit Negation | 1.2528 | 0.6976 | 1.796 | 0.07251 |
| Unfamiliar/Negative: Social Learning | 15.1418 | 3237.1463 | 0.005 | .9962 |
| Unfamiliar/Negative: Explicit Negation | 15.7740 | 3237.1463 | 0.005 | .9961 |

**Table S4.** **Model_0_ , Model_1_ and Model_2_ for the *Target Fixated trials****.* Estimated (fixed) regression coefficients with their standard errors, *z*- and *p*-values (* *p* < .05).

|  |  |  |  |  |
| --- | --- | --- | --- | --- |
| **Null-hypothesis Model_0_: RedundantSaccades ~ Task + (1\| Participant)** | | | | |
|  | Estimate | *SE* | *z* | *p* |
| (Intercept) | -2.8001 | 0.7355 | -3.807 | <.0001* |
| Social Learning | -0.2328 | 1.0299 | -0.226 | .8211 |
| Explicit Negation | -0.1412 | 1.0880 | -0.130 | .8967 |
|  |  |  |  |  |
| **Main Effect Model_1_: RedundantSaccades ~ Task + PromptType + (PromptType\| Participant)** | | | | |
|  | Estimate | *SE* | *z* | *p* |
| (Intercept) | -3.2267 | 0.9738 | -3.314 | <.001* |
| Unfamiliar/Negative | 0.9650 | 0.8779 | 1.099 | .2716 |
| Social Learning | -0.6099 | 1.0572 | -0.577 | .5639 |
| Explicit Negation | -0.3258 | 1.0031 | -0.325 | .7453 |

| **Interaction Model_2_: RedundantSaccades ~ Task + PromptType + PromptType:Task + (PromptType\| Participant)** | | | | |
| --- | --- | --- | --- | --- |
|  | Estimate | *SE* | *z* | *p* |
| (Intercept) | -3.57551 | 1.39795 | -2.558 | <.05* |
| Prompt Type | 1.39552 | 1.48192 | 0.942 | .3463 |
| Social Learning | 0.14433 | 1.80195 | 0.080 | .9362 |
| Explicit Negation | 0.05658 | 2.02019 | 0.028 | .9777 |
| Unfamiliar/Negative: Social Learning | -1.06061 | 2.09394 | -0.507 | .6125 |
| Unfamiliar/Negative: Explicit Negation | -0.457 | 2.1629 | -0.211 | .8327 |

**Table S5.** **Model_0_ , Model_1_ and Model_2_ for the *Distractor Fixated trials****.* Estimated (fixed) regression coefficients with their standard errors, *z*- and *p*-values (* *p* < .05).

**Supplementary Note 4: exploratory analyses of the double-check and redundant saccades with excluded participants.**

To probe the robustness of toddlers' double-check and redundant saccades analyses, we also ran an exploratory analysis including the seven participants who were excluded from the analysis since they lacked valid trials in at least one of the four conditions. The Bayesian analysis of double-checks (see the Main Text page 7) on *Target-Fixated trials* (n = 232)revealed that the data are 4.1*10^2^ times more likely under H1 than H0: in the *Target-Fixated trials* the double-check is more probable in the *Unfamiliar/Negative* condition than in the *Familiar/Positive* one, (*Unfamiliar/Negative*, *M* = 58%, *SD* = 33%; *Familiar/Positive* = 21%, *SD* = 27%). Furthermore, the analysis revealed that the data are 2.1*10^2^ times more likely under H1 than H2: that is, there is no interactions and the three tasks resulted in a similar pattern of results. This result was confirmed by an ANOVA (Type II Wald chi square tests, r package car) that detected a main effect of Prompt Type (X^2^ (1, N = 232) = 25.78, p < .0001), a main effect of Task (*Word-learning*, *M* = 23%, *SD* = 30%; *Social-Learning, M* = 52%, *SD* = 32%; *Explicit-Negation*, *M* = 46%; *SD* = 39%; X^2^ (2, N = 232) = 14.55, p < .001), but no interaction effect (X^2^ (2, N = 232) = 0.18, p > .05). Planned follow-up pairwise comparisons (package r emmeans) corroborated the effects of Prompt Type in each Task: *Word-learning (Unfamiliar, M* = 39%, *SD* = 36%; *Familiar*,  *M* = 9%, *SD* = 16%; p < .01 ), *Social-learning (Unfamiliar, M* = 70%, *SD* = 25%; *Familiar*,  *M* = 32%, *SD* = 26%, p < .01), *Explicit-Negation (Unfamiliar, M* = 65%, *SD* = 33%; *Familiar*,  *M* = 26%, *SD* = 36%; p < .01).

Next, we considered the scanning pattern that occurred on both *Distractor-Fixated Familiar/Positive trials* and *Distractor-Fixated Unfamiliar/Negative trials*. On these trials (n = 184), the Bayesian analysis revealed that the data are 2.5*10^3^ times more likely under H0 than H1: that is, on *Distractor-Fixated trials*, there was no effect of Prompt Type on the probability of observing double-checks (*Unfamiliar/Negative*: *M* = 3%, *SD* = 16%; *Familiar/Positive*: *M* = 4%, *SD* = 17%). The data were 63 times more likely under H1 than H2. There were no interactions – the three tasks yielded a similar pattern of results.

We also compared the probability of redundant saccades in the *Distractor-Fixated Familiar/Positive trials* with the *Unfamiliar/Negative* ones (see Main Text page 9). By our hypothesis, there should be no difference in the probability of redundant saccades in the two conditions. Indeed, the analysis revealed that the data are 1.2*10^2^ times more likely under H0 than H1: in the *Distractor-Fixated trials*, there is no effect of Prompt Type on the probability of redundant saccades (*Unfamiliar/Negative*: *M* = 10%, *SD* = 26%; *Familiar/Positive*: *M* = 7%, *SD* = 20%). Furthermore, the analysis revealed that the data are 181*10^2^ times more likely under H1 than H2: there is no interaction, and the three tasks resulted in a similar pattern of results. This argues that rejection of the competitor object increases the certainty that the participant has that the target object is the correct target – up to a level equivalent to the Familiar/Assertion prompts.

Finally, we also compared the probability of redundant saccades in the *Target-Fixated trials* by the same analysis. The analysis revealed that the data are 4.7*10^5^ times more likely under H1 than H0: in the Target Fixated trials, redundant saccades are *more likely* in the *Familiar/Positive* condition than in the *Unfamiliar/Negative* one (*Unfamiliar/Negative*: *M* = 2%, *SD* = 10%; *Familiar/Positive*: *M* = 27%, *SD* = 31%). Furthermore, the analysis revealed that the data are 1.5*10^2^ times more likely under H1 than H2: there is no interactions and the three tasks resulted in a similar pattern of results.

**Supplementary Note 5: exploratory analysis of the double-check probability with all trial types and compared at once.**

To further probe the robustness of toddlers' double-check analyses, we also ran an exploratory analysis including double-check data from all tasks and all four trial types. Trial types were coded as a single four levels factor TrialType (Target-Fixated.Familiar/Positive; Target-Fixated.Unfamiliar/Negative; Distractor-Fixated.Familiar/Positive; Distractor-Fixated.Unfamiliar/Negative). The double-check data were fit three models. The "null-hypothesis" model *Double-Check ~ Task + (1| Participant)* had no effect for Trial Type. The “MainEffects” model *Double-Check ~ Task + TrialType + (PromptType| Participant)* had the effect for Trial Type. The "interaction" model *Double-Check ~ Task + Trial Type + Trial Type:Task + (PromptType| Participant)* also included the interaction between Trial Typee and Task.

*T*he null-hypothesis Model**_0_** scored BIC = 419.57, the main effect Model**_1_** scored BIC = 369.25, and the interaction Model**_2_** scored BIC = 395.14. A comparison between the null-hypothesis Model**_0_** and the main effect Model**_1_** yielded a Bayes factor BF10 = 8.4*10^10^, and a comparison between the main effect Model**_1_** and the interaction Model**_2_** yielded a Bayes factor BF12 = 4.1*10^5^ indicating strong evidence for the main effect model (Jeffreys, 1985).

This result was confirmed by an ANOVA (Type II Wald chi-square tests, r package car) that detected a main effect of TrialType (X^2^ (3, N = 379) = 40.58, p < .0001), a main effect of Task ( X^2^ (2, N = 379) = 10.9, p < .01), but no interaction effect (X^2^ (6, N = 379) = 1.6, p > .05). Planned follow-up pairwise comparisons (package r emmeans) corroborated that the double-check was more likely in the *Target-Fixated Unfamiliar/Negative trials (M* = 57%, *SD* = 33%) than in the *Target-Fixated Familiar/Positive trials* (*M* = 23%, *SD* = 28%; p < .0001), *Distractor-Fixated Unfamiliar/Negative trials* (*M* = 2%, *SD* = 10%; p < .0001), and *Distractor-Fixated* *Familiar/Positive trials* (*M* = 4%, *SD* = 17%; p < .0001).

In summary, this analysis confirmed that in the *Target-Fixated Unfamiliar/Negative trials*, toddlers' were *more* likely to perform a single double-check before pointing in response and that this pattern was not affected by the task content/domain. These results confirm the prediction made by the domain-general Disjunctive Inference.

**
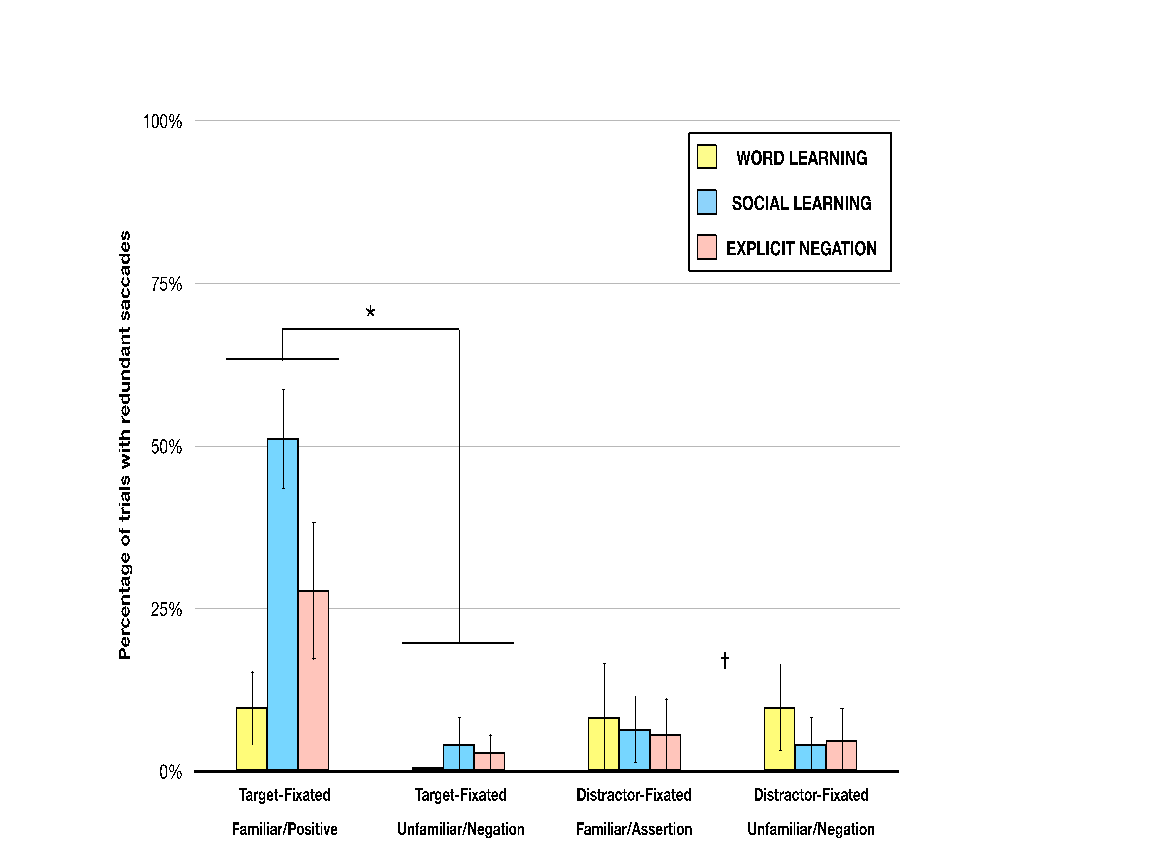
**

**Supplementary Fig. 1. Percentage of trials with redundant saccades as a function of prompt type and object fixated.** Bars reflect percentage of trials with one or more saccades above the ones predicted by a confident choice based on memory alone (*Familiar/Positive trials*) or the Disjunctive Inference (*Unfamiliar/Negation trials*). Across all three tasks, the percentage of trials with redundant saccades was NOT higher when toddlers were fixating the competitor and heard an *Unfamiliar/Negation prompt*, consistent with the Disjunctive Inference. Bayes Factor tests reveal that an effect of prompt type on *Target-Fixated trials* is >50 times *more* likely than no effect (*), and that an effect of prompt type on *Distractor-Fixated trials* is >50 times *less* likely than no effect (✝). Redundant saccades are likely to index uncertainty, the relatively low percentage of redundant saccades in the Unfamiliar/Negation trials suggests that toddlers' Disjunctive Inference resulted in a confident decision.

**Supplementary References**

Jeffreys, H. (1985). *Theory of probability* (3rd ed). Clarendon press.

Schwarz, G. (1978). Estimating the Dimension of a Model. *The Annals of Statistics*, *6*(2). https://doi.org/10.1214/aos/1176344136

Wagenmakers, E.-J. (2007). A practical solution to the pervasive problems ofp values. *Psychonomic Bulletin & Review*, *14*(5), 779–804. https://doi.org/10.3758/BF03194105
